# Subject-Specific Increases in Serum S-100B Distinguish Sports-Related Concussion from Sports-Related Exertion

**DOI:** 10.1371/journal.pone.0084977

**Published:** 2014-01-08

**Authors:** Karin Kiechle, Jeffrey J. Bazarian, Kian Merchant-Borna, Veit Stoecklein, Eric Rozen, Brian Blyth, Jason H. Huang, Samantha Dayawansa, Karl Kanz, Peter Biberthaler

**Affiliations:** 1 Department of Trauma Surgery, Klinikum der Universität München, Ludwig-Maximilians Universität, München, Germany; 2 Department of Emergency Medicine, University of Rochester Medical Center, Rochester, New York, United States of America; 3 Department of Neurosurgery, Klinikum der Universität München, Ludwig-Maximilians Universität, München, Germany; 4 Department of Athletics and Recreation, University of Rochester, Rochester, New York, United States of America; 5 Department of Neurosurgery, University of Rochester Medical Center, Rochester, New York, United States of America; 6 Department of Trauma Surgery, Technical University of Munich, München, Germany; University of Cincinnati, United States of America

## Abstract

**Background:**

The on-field diagnosis of sports-related concussion (SRC) is complicated by the lack of an accurate and objective marker of brain injury.

**Purpose:**

To compare subject-specific changes in the astroglial protein, S100B, before and after SRC among collegiate and semi-professional contact sport athletes, and compare these changes to differences in S100B before and after non-contact exertion.

**Study Design:**

Longitudinal cohort study.

**Methods:**

From 2009–2011, we performed a prospective study of athletes from Munich, Germany, and Rochester, New York, USA. Serum S100B was measured in all SRC athletes at pre-season baseline, within 3 hours of injury, and at days 2, 3 and 7 post-SRC. Among a subset of athletes, S100B was measured after non-contact exertion but before injury. All samples were collected identically and analyzed using an automated electrochemiluminescent assay to quantify serum S100B levels.

**Results:**

Forty-six athletes (30 Munich, 16 Rochester) underwent baseline testing. Thirty underwent additional post-exertion S100B testing. Twenty-two athletes (16 Rochester, 6 Munich) sustained a SRC, and 17 had S100B testing within 3 hours post-injury. The mean 3-hour post-SRC S100B was significantly higher than pre-season baseline (0.099±0.008 µg/L vs. 0.058±0.006 µg/L, p = 0.0002). Mean post-exertion S100B was not significantly different than the preseason baseline. S100B levels at post-injury days 2, 3 and 7 were significantly lower than the 3-hour level, and not different than baseline. Both the absolute change and proportional increase in S100B 3-hour post-injury were accurate discriminators of SRC from non-contact exertion without SRC (AUC 0.772 and 0.904, respectively). A 3-hour post-concussion S100B >0.122 µg/L and a proportional S100B increase of >45.9% over baseline were both 96.7% specific for SRC.

**Conclusions:**

Relative and absolute increases in serum S100B can accurately distinguish SRC from sports-related exertion, and may be a useful adjunct to the diagnosis of SRC.

## Introduction

Sports-related concussions (SRC) are common in both the United States and Germany. In the US, the Centers for Disease Control and Prevention estimates that there are 1.6–3.8 million SRC per year [1], with the highest concussion rates found among contact sports such as ice hockey, football, soccer, and basketball [2].While there are no similar cumulative figures for Germany, a recent epidemiological survey found the overall incidence of traumatic brain injuries (TBI) to be 330/100,000 inhabitants and that 6.3% of these were due to sports [3], [4]. With the current German population at just over 81.5 million, that translates into over 268,000 sports TBIs per year.

After a concussion, most athletes recover within 7–10 days [5], but up to 10% have post-concussion symptoms such as headache and dizziness lasting longer than 7 days [6], [7]. In addition, multiple concussions are thought to be linked to the early onset of neurodegeneration, known as chronic traumatic encephalopathy [8]. Existing concussion management guidelines are designed to mitigate these risks and involve primarily rest [5]. However, the effect of these guidelines on reducing long-term disability depends entirely on recognizing that a concussion has occurred in the first place. While sideline tools such as SAC [9], SCAT 2 [5], and SCAT 3 [10] are designed to assist coaches and certified athletic trainers in identifying the mental status changes (loss of consciousness, amnesia, confusion) that are the hallmark of this injury, unrecognized or unreported concussions are still quite common, occurring in over half of injured high school football players queried via a confidential survey [11]. An objective parameter that can be easily and rapidly measured on-site could optimize the identification and clinical management of concussed athletes.

Serum S100B has emerged as a potential candidate in this regard. S100B is a 21 kDa dimeric protein expressed primarily by brain astrocytes and belongs to a multigenic family of calcium-binding proteins [12]–[14]. Its primary clinical role in concussion management has been to help identify the approximately 5% of concussion patients that have intracranial hemorrhage on head CT scan. Although S100B's specificity for abnormal head CT is low (40%), its high sensitivity (99%) [15]–[17] has led to its adoption as a clinical tool to screen for head injured patient requiring a head CT scan (pre-head CT screen) in several countries in Europe and Asia.

Enthusiasm for the use of S100B to aid in the diagnosis of concussion, however, has been tempered by significant overlap between levels in healthy individuals and in athletes who have had a concussion. Using cutoffs derived from comparing these two groups, only very high S100B levels—rarely seen after concussion–are considered abnormal, making this test appear clinically useless in a sports setting. However, small but significant post-concussion increases can be revealed by comparing S100B values not among groups, but in *individual athletes* before injury to that after injury. This kind of study is expensive and labor-intensive to perform because one cannot predict which athletes will get injured during a season; serum must be obtained from several hundred athletes in order to accrue pre and post-concussion samples in 20–30 athletes.

A second problem for using S100B clinically is the observation that serum levels rise not only after concussion but also after a game in which no concussion was observed [18]–[21]. Explanations for this observation range from exertion-related release of small amounts of S100B from melanocytes, chondrocytes and adipocytes [12], to occult or unreported brain injury, or a combination of the two. In order to separate the effect of these two processes on S100B, one needs to compare S100B increases in concussed athletes to that in a group of athletes undergoing exertion but not body contact in whom the risk of even occult brain injury is very low.

In an effort to unlock the diagnostic potential of S100B, we sought to compare subject-specific changes in S100B before and after concussion among collegiate and semi-professional athletes. Our secondary aim was to compare these changes to differences in S100B before and after non-contact exertion. By using an intra-subject, before-and-after study design, we minimized the effect that individual-level variation in baseline S100B levels has on obscuring group-level post-concussion increases. In addition, by examining S100B changes in a reference group not undergoing sports-related contact, we sought to minimize the probability that S100B elevations in the comparison group are due to occult brain injury.

## Methods

We performed a longitudinal cohort study of contact sport athletes in Munich and Rochester between 2009 and 2011.

### Ethics Statement

The study was approved by the ethics committees of the Ludwig Maximilians University in Munich, Germany and at the Rochester Institute of Technology and University of Rochester in Rochester, NY, USA. Voluntary participation was solicited through communication with athletic directors, coaches, and athletes. All participating athletes completed written informed consent forms before inclusion in the study, which described the purpose, objective, and details of the testing and research participation.

### Inclusion and Exclusion Criteria

Athletes were eligible for inclusion if they were ≥18 years of age and active participants in contact sport teams affiliated with the Ludwig Maximilians University in Munich, Germany, or with the University of Rochester or Rochester Institute of Technology in Rochester, New York.

The University of Rochester participates in several National Collegiate Athletic Association (NCAA) Division III sports including the following contact sports: football, soccer, and basketball. In addition, the University of Rochester provides orthopedic and trauma care to hockey players at Rochester Institute of Technology (men participate in NCAA Division I and women, at the time of the study, in Division III). Athletes from these teams were eligible to participate. Although German universities don't participate in US-style collegiate athletic organizations, the Ludwig Maximilians University provides orthopedic and trauma care to the Straubing Tigers Ice Hockey Club which is a semi-professional ice hockey team that currently plays in the Deutsche Eishockey Liga (*German National Ice Hockey League*). Athletes from this team were eligible to participate in the current study.

Athletes who sustained a moderate or severe TBI (determined by a hospital GCS of 3–12) were excluded from the study. Athletes who sustained a concussion within two weeks of baseline (ie: during the off-season or during a non-sport activity) were also excluded. History of prior head injury was determined by self-report using a previously validated survey tool[22].

### Serum Sampling

Among Rochester athletes, baseline samples were collected on all participating athletes but analyzed for S100B only in those who subsequently developed concussion. Samples were collected within 3 hours of SRC and at days 2, 3 and 7 post-concussion. Among Munich athletes, baseline samples were collected and analyzed for S100B in all participants, then again after a period of exertion (but before injury), and finally on the subset who suffered a concussion, also within 3 hours of injury **(** **Table 1** **)**. Exertion consisted of non-contact ice-hockey skating drills. Post exertion testing was limited to Munich athletes principally for reasons of convenience. The Straubing Tigers had the largest number of participating athletes of any single participating team, as well and a regular practice schedule made in advance. These factors facilitated post-exertion serum sample with a minimum of study personnel. Because members of Straubing Tigers were frequently on the road, post-concussion serum was not obtained from Munich athletes beyond the 3-hour time point.The study group thus consisted of athletes with S100B samples obtained before and after concussion, and the comparison group consisted of athletes with S100B samples obtained before and after non-contact exertion.

**Table 1:** Study overview.

Munich Athletes (n)		Rochester Athletes (n)
30	Pre-season (resting) S100B	16
30	Post-exertion S100B	0
6	Concussion	16
6	3-hours Post-Concussion S100B	11
0	2 days Post-Concussion S100B	16
0	3 days Post-Concussion S100B	15
0	7 days Post-Concussion S100B	16

Athletes were considered concussed if they had an injury witnessed by an on-field coach or certified athletic trainer meeting the definition of concussion defined by the Sport Concussion Assessment Tool 2 (SCAT2) [5]. This definition consists of an injury resulting in any one or more of the following: symptoms (such as headache), physical signs (such as unsteadiness), impaired brain function (such as confusion), or abnormal behavior.

Methods for obtaining and handling serum were identical at both sites. Four milliliters of venous blood was drawn into sterile Vacuatiner serum separator tubes and immediately placed on 0°C ice. Within 60 minutes, the blood was centrifuged (3000 rpm, 10 minutes), and the serum separated and stored at −80°C until sample analysis.

### Serum S100B Measurement

Serum S100B concentrations were determined by electrochemoluminometric immunoassay (ELECSYS® S100, ROCHE Diagnostics, Mannheim, Germany) with a detection limit of 0.005–39 µg/L, according to the manufacturer's instructions.. Briefly, two monoclonal antibodies directed against the beta-chain of the S100 dimer were inclubated with 20 µl of sample for 9 minutes. Streptavidin-coated microparticles were added, and the mixture was incubated for an additional 9 minutes in order to form a solidified immunocomplex. The reaction mixture was transferred to a measurement cell where the beads were magnetically captured on an elctrode surface, and unbound components were removed by washing. A defined voltage was applied to the electrode to initiate the electrochemiluminescent reaction and the resultant light emission was measured using a photomultiplier. Serum S100B concentrations were reported as μg/L of serum and reported as mean values ± SD. Reference range of S100B is 0.02 to 0.15 µg/L [23].

### Analysis

Serum S100B concentrations before and after SRC, as well as before and after non-contact exertion, were compared using paired t-tests. Changes in S100B concentrations among all concussed athletes were compared using paired and unpaired t-tests. Receiver operating characteristic (ROC) analyses were performed to evaluate S100B's classification accuracy for SRC [24]. In these analyses, S100B levels within 3 hours of concussion were compared to S100B levels after non-contact exertion. We examined the ROC of the post-SRC or post-exertion S100B value as well as the proportional change in post-concussion/exertion S100B relative to preseason (baseline). The proportional change was defined as [(post-exertion or 3-hour post-SRC S100B – baseline S100B)/baseline S100B]. Analyses were performed using SAS®Software Version 9.3 (SAS Institute Inc., Cary, NC, USA) and GraphPad Prism Version 5.02 for Windows (GraphPad Software, La Jolla California USA). A p-value of ≤0.05 was considered statistically significant.

This study was designed to to detect clinically-relevant increases in S100B. Because the sample size was fixed by the number of concussed subjects (46), we report here the power this sample size had to detect clinically relevant S100B increases. Cliniclaly-relevant increases were defined based on prior studies reporting baseline S100B levels in athletes ranging from 0.11–0.22 ug/L (SD range 0.04–0.04 ug/L) that increased by 36–64% after exertion[20], [25]. Thus, assuming a Type I error of 0.05, mean baseline S100B level of 0.11 ug/L, and SD in mean baseline S100B of 0.05 ug/L, 46 sujbects provides 96% power to detect an increase of 25% or greater in mean baseline S100B levels.

## Results

Thirty Munich athletes agreed to participate, providing baseline and post-exertion serum samples. All of these samples were analyzed for S100B. Six of these athletes suffered a SRC during the study period **(** **Table 1** **)**. A total of 306 Rochester athletes agreed to participate, providing baseline (but not post-exertion) serum samples. Sixteen of these athletes suffered a SRC during the study period. Only the serum samples from the 16 concussed Rochester athletes were analyzed for S100B at baseline and after SRC. The initial, within-3-hour serum sample was not obtained in five of these 16 athletes because the SRC occurred during a game outside the Rochester area; however, subsequent samples were obtained at the later time points.

Forty-six athletes (30 from Munich and 16 from Rochester) had baseline samples, as well as post-exertion and/or post-SRC samples, analyzed for S100B. The mean age of all athletes was 25.4 years and 89% were male **(** **Table 2** **)**. The mean age of Munich athletes was significantly greater than the mean age of Rochester athletes (28.4±0.82 years vs. 19.8±0.34 years, p<0.001). The mean baseline S100B concentration among Munich athletes was not significantly different than the mean baseline concentration among Rochester athletes (0.070±0.03 µg/L vs. 0.068±0.04 µg/L, p = 0.85).

**Table 2:** Subject characteristics.

Characteristic	Munich Athletes (n = 30)	Rochester Athletes (n = 16)	Combined Cohort (n = 46)
Age, Mean Years (SD)	28.4 (4.5)	19.8 (1.4)	25.4 (5.5)
Male, N (%)	30 (100)	11(68.8)	41 (89.1)
Race			
Caucasian, N (%)	30 (100)	14 (87.5)	44 (95.7)
African American/European, N (%)	0	2 (12.5)	2 (4.3)
Sport			
Ice Hockey, N (%)	30 (100)	4 (25)	34 (73.9)
Soccer, N (%)	0	3 (18.8)	3 (6.5)
Football, N (%)	0	8 (50)	8 (17.4)
Basketball, N (%)	0	1 (6.2)	1 (2.2)
Baseline S100B, mean μg/L (SD)*	0.070 (.03)	0.068 (.04)	0.070 (.03)

T-test to assess difference between Munich and Rochester cohorts at baseline, p = 0.85.

**Figure 1:**
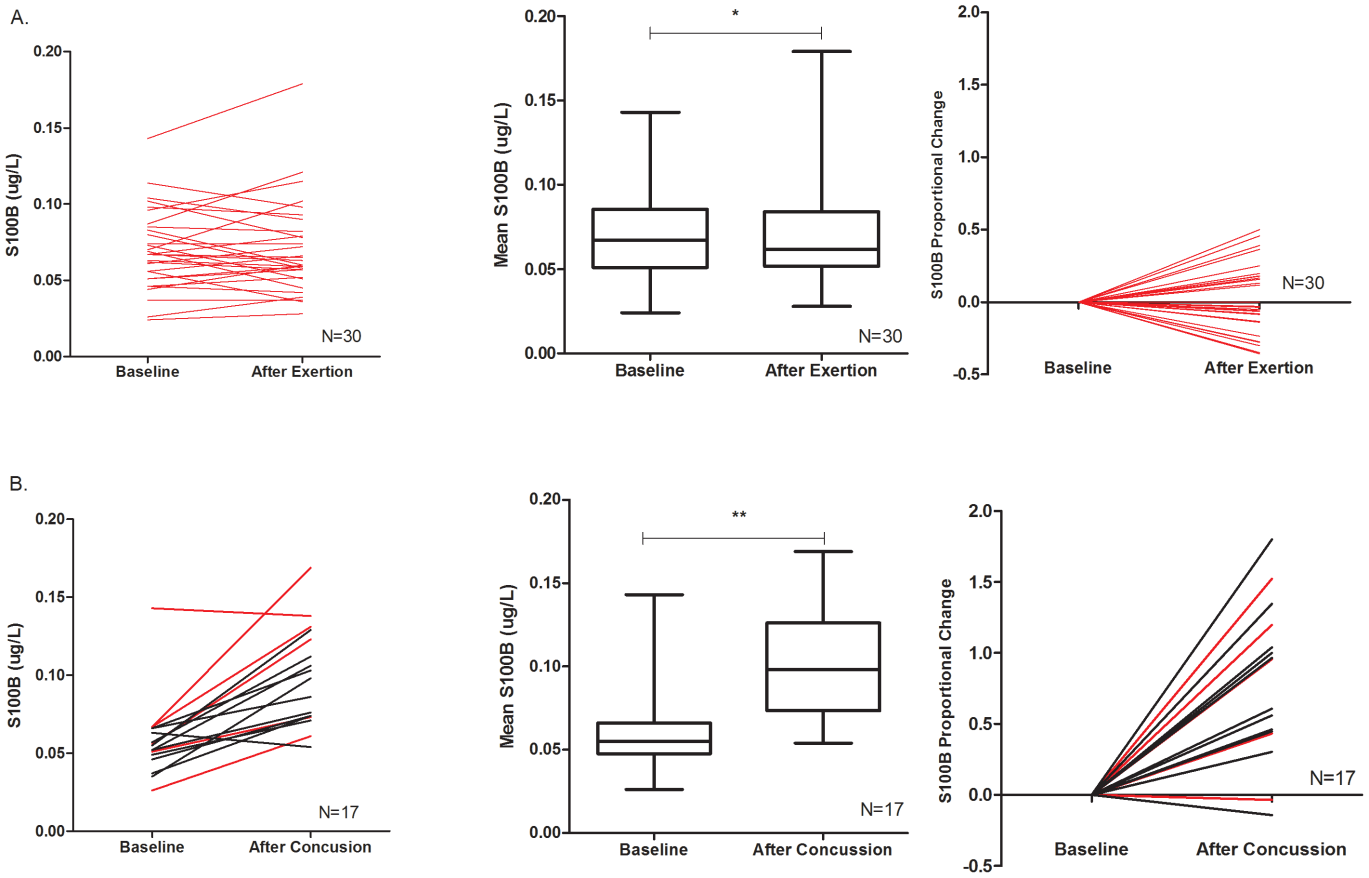
S100B Changes after non-contact exertion and sports-related concussion. **A**: Absolute (Left) and proportional (right) changes in S100B levels after non-contact exertion among individal athletes. The proportional change is defined as (post-exertion or 3-hour post-SRC S100B – baseline S100B)/baseline S100B (range: −35% to +50%; mean proportional change  = +2.7%). The mean post-exertional S100B concentration (0.071±0.03 µg/L) was not significantly different from mean baseline (0.070±0.03 µg/L, *p = 0.87, Middle). **B**: Absolute (Left) and proportional (right) changes in S100B levels within 3 hours of SRC among individual athletes. The mean post-SRC S100B concentration within 3 hours of concussion (0.099±0.008 µg/L) was significantly higher than that at baseline (0.058±0.006 µg/L, **p = 0.0002, Middle). Proportional change (right) ranged from −14% to +180%; mean proportional change +81.2%). Munich athletes are designated in red, Rochester athletes in black.

Among the 30 Munich athletes who underwent post-exertion S100B testing, there was no difference in mean post-exertion S100B level compared to baseline (0.071±0.03 µg/L vs. 0.070±0.03 µg/L, p = 0.87) **(** **Figure 1** **)**. Proportional post-exertion changes in S100B ranged from −35 to +50%. The mean proportional post-exertion S100B change was +2.7%.

**Figure 2:**
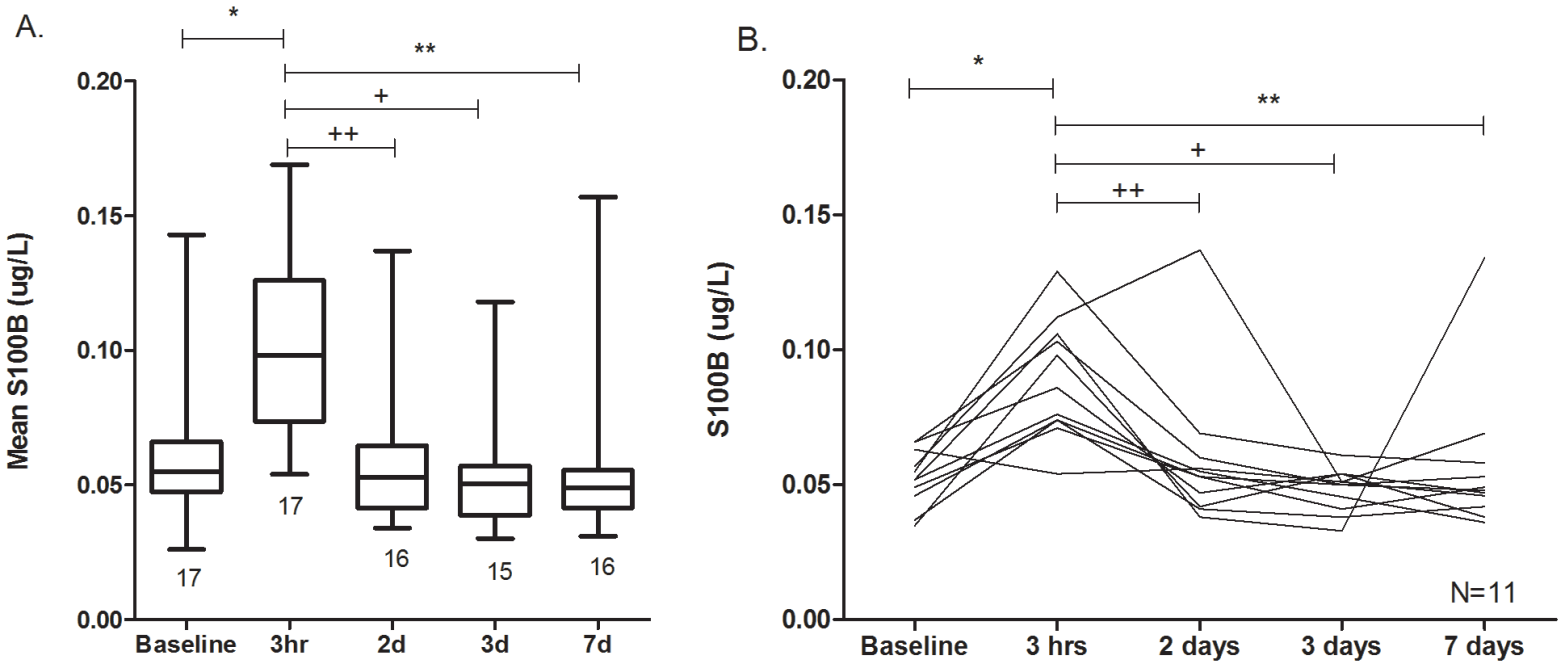
Longitudinal changes in S100B before and after sports-related concussion. **A**: Mean changes in S100B levels among all concussed athletes at baseline and at four time points after injury. The number of athletes included in determining the mean at each time point is indicated below each box plot. The mean S100B concentration within 3 hours of SRC (0.099±0.032 µg/L) was significantly higher than that at baseline (0.058±0.025 µg/L, *p<0.0001) using a paired t-test. The 3 hour S100B level was also higher than that at 2 days (0.059±0.03 µg/L, ^++^p = 0.0004), 3 days (0.052±0.02 µg/L, ^+^p<0.0001), and 7 days (0.059±0.03 µg/L, **p = 0.001) post-SRC using unpaired t-tests. **B**: Longitudinal changes in S100B levels among concussed athletes who had baseline and all four post-SRC S100B measurements. All 11 of these athletes were from the Unviersity of Rochester. The mean S100B level within 3 hours of SRC (0.099±0.032 µg/L) was significantly higher than that at baseline (0.058±0.025 µg/L, *p<0.0001), and higher than that at 2 days (0.059±0.027 µg/L, ++p = 0.0047), 3 days (0.048±0.008 µg/L, +p = 0.0002), and 7 days (0.056±0.027 µg/L, **p = 0.0058) post-SRC using paired t-tests.

During the study period, 22 athletes (17 male and 5 female; 16 from Rochester and 6 from Munich) suffered a SRC, of which serum samples were collected within 3-hours of injury from 17 individuals (11 from Rochester and 6 from Munich). None of these concussions were accompanied by other extracranial fractures or lacerations. Among these 17 athletes, the mean S100B level within 3 hours of injury was significantly higher than baseline (0.099±0.008 µg/L vs. 0.058±0.006 µg/L, paired t-test p<0.0001; **Figure 1**). Proportional post-SRC changes in S100B ranged from −14 to +180%. The mean post-SRC S100B change was +81.2%. Longitudinal S100B levels measured post-injury days 2, 3 and 7 were not significantly different than baseline, although they were all significantly lower than the 3-hour post injury level **(** **Figure 2** **)**.

**Figure 3:**
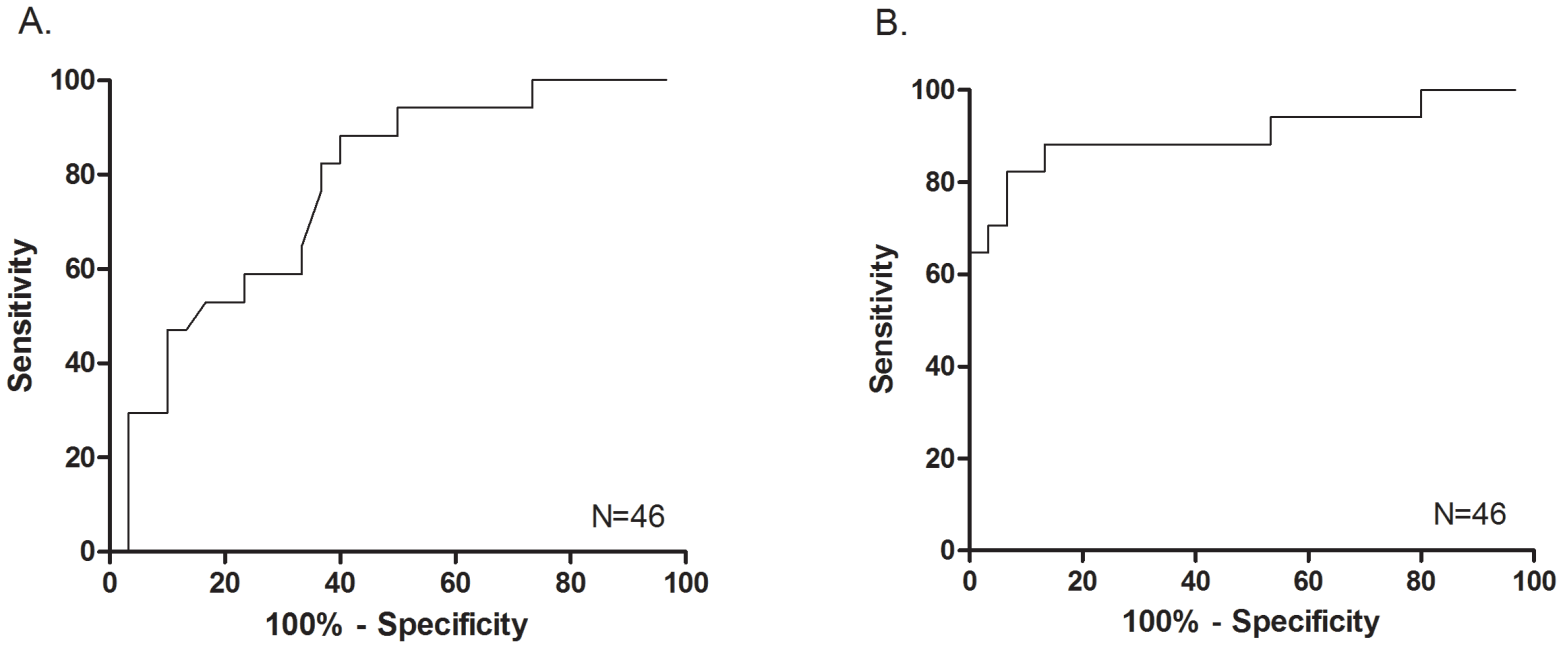
Diagnostic accuracy of S100B for sports-related concussion. A: The ROC using the 3-hour post-SRC value or the post-exertion S100B value revealed an AUC of 0.772 (95% CI: 0.64, 0.91). B: The AUC using the proportional increase in post-SRC/exertional S100B level over baseline was 0.904 (95% CI: 0.80, 1.0).

The ROC using the absolute value 3-hour post-injury/exertional S100B value revealed an AUC of 0.772 (95% CI: 0.64, 0.91). However the AUC using the proportional increase in post-injury/exertional S100B level over baseline was higher (0.904, 95% CI: 0.80, 1.0; **Figure 3**). Cutoff values that maximize sensitivity and specificity for SRC diagnosis are shown in **Table 3**. A 3-hour post-concussion S100B level of >0.122 µg/L and a proportional S100B increase of >45.9% over baseline were both 96.7% specific for concussion.

**Table 3:** Concussion classification using S100B.

	Cutoff	Sensitivity (95% CI)	Specificity (95% CI)
3-hour S100B	0.0605 µg/L^†^	94.1% (71,100)	50.0% (31, 69)
	0.122 µg/L^‡^	29.4% (10, 56)	96.7% (83,100)
			
Proportional Change	−3.5%^†^	94.1% (71,100)	46.7% (28, 66)
in 3-hour S100B relative	45.9%^‡^	70.6% (44, 90)	96.7% (83, 100)
to baseline			

Cutoff values shown are those that maximize sensitivity (†) and specificity (‡).

## Discussion

The current approach to diagnosing SRC is complicated by the subjective nature of the current sideline assessment tools as well as by symptom minimization among athletes motivated to remain in the game [11]. The lack of an objective and accurate diagnostic aid hinders efforts to minimize both the short and long-term consequences of SRC.

In the current study, we have demonstrated that elevated S100B measured within 3 hours of injury is an accurate discriminator of SRC from sport-related exertion. Classification accuracy is greatly enhanced if the post-injury value is compared to a pre-season baseline. This will come as little surprise to those accustomed to comparing an injured athlete's post-concussion cognitive test scores to their pre-season scores. Unlike tests of cognition, however, measurements of serum S100B are less likely to be affected by stress, sleep deprivation, and pain. Moreover, baseline levels can't be manipulated by athletes trying to minimize the effects of potential future concussions. On the other hand, unlike cognitive performance, which can remain abnormal for weeks after concussion, serum S100B levels are cleared rapidly [26]; in our study S100B levels were back to baseline by day 2 after SRC. Thus, while the initial 3 hour level may be a useful adjunct to the diagnosis of SRC, subsequent S100B levels can't be used as a diagnostic aid, and will not be helpful in determining when an athlete is recovered and ready to return to contact sports.

Based on our preliminary findings, an S100B level of >0.122 µg/L or a proportional S100B increase of >45.9% over baseline measured within 3 hours of injury is quite specific for SRC, while a level <0.0605 µg/L or a proportional decrease of >3.5% essentially rules it out. Six concussed athletes had serum drawn within 15 minutes of injury (5 of 6 were elevated) suggesting that sampling could be done on the sideline, relatively soon after injury. The prompt measurement of S100B is ideal, given the relatively short biological half-life of S100B. Pending confirmation in larger studies, these results suggest that S100B could be used to aid in the sideline diagnosis of SRC.

However, several practical issues remain. Although used clinically in Germany, S100B testing has not been approved by the FDA for use in the US. In addition, there is currently no portable, point of care assay for S100B measurement, making sideline testing problematic. The Roche Elecsys® analyzer used in the current study is the size of a large desktop printer and requires a centrifuged venous blood sample. Point of care test devices that require a finger stick sample of blood are, however, currently under development [27]. The rollout of these devices would allow athletic trainers to collect baseline as well as post injury S100B levels in a training room or athletic field. Baseline finger stick S100B testing could become integrated into the required pre-participation physical exam that is part of collegiate and secondary sports participation in both the US and Germany. This idea may not be so implausible given the 2010 NCAA regulations in the US requiring pre-participation blood testing for the sickle cell trait[28]. The effort required to overcome these obstacles would be more than offset by the potential benefits of accurately diagnosing and managing an injury to the brain affecting millions of athletes around the world.

While our study has many strengths, including a before-and-after design and a comparison group with a very low risk of occult brain injury, there are several limitations. The primary results involved combining data from two seemingly distinct cohorts; one from Munich and one from Rochester. Although athletes in both cohorts consisted of young adults playing their sport at a highly competitive level, the cohorts differed with respect to age, gender, and sport. They also differed with respect to concussion prevalance; 20% for the Munich athletes and <1% for the Rochester athletes. This difference may be due in part to the increased concussion risk associated with semi-professional ice hockey but more likely reflects differences in play time. Munich athletes played 8 months a year while the collegiate athletes in Rochester played for only 2–3 months. Because each player served as their own control, these differences were unlikley to bias the main results. Moreover, the mean baseline S100B levels are nearly identical in the two cohorts, as were the patterns of post-injury S100B change **(** **Table 2** **and** **Figure 1** **)**. These observations underscore the legitimacy of analyzing the groups together. Finally, all of the SRCs in our study were isolated injuries to the head, without the potentially confounding effects of extracranial injuries on S100B elevations. Although others have demonstrated that the contribution of extracranial S100B to overall serum levels is negligible [29], our results should be applied with caution to SRCs accompanied by significant extracrainal injuries such as fractures.

In summary, current sideline diagnosis of SRC relies on athlete self-report or trainer/coach recognition using SAC, SCAT 2, or SCAT 3. Despite this, unrecognized or unreported concussions are still quite common. Our results suggest that serum S100B measured within 3 hours of a sports-related injury can accurately distinguish SRC from sports-related exertion. The accuracy of this prediction is enhanced if the post-injury level is compared to a pre-season baseline level, much in the same way that cognitive test scores are currently interpreted. Serum S100B >0.122 µg/L or a proportional S100B increase of >45.9% over baseline are highly specific (96.7%) for SRC. These results suggest that serum S100B may be a useful adjunct to the diagnosis of SRC.
